# Pre-treatment of *Cucurbita maxima* ‘Hokkaido orange’ by *Viscum album* aqueous extracts in search of allelopathic potential

**DOI:** 10.1038/s41598-024-65918-0

**Published:** 2024-06-28

**Authors:** Oleksandra Strashok, Monika Ziemiańska, Marta Czaplicka, Vitalii Strashok

**Affiliations:** 1 Wroclaw University of Environmental and Life Sciences, Wrocław, Poland; 2 National University of Life and Environmental Sciences of Ukraine, Kyiv, Ukraine

**Keywords:** Plant development, Plant physiology

## Abstract

*Viscum album* L. (VA) is a unique plant with regard to its biological content. It is rich in many different metabolites with high potential in various spheres of human activity. We conducted a pilot study with 5 VA aqueous extracts of different host-tree species for pre-sowing treatment of *Cucurbita maxima* ‘Hokkaido orange’ seeds. We set the following objectives consisting of hypotheses (1) *H01* is based on different effects of tested VA extracts depending on host trees and time of pre-treatment; (2) *H02* focuses on the allopathic properties of the tested extracts affecting the plant growth and development by dose–response relationship; (3) *A01* considers highly biologically active compounds of VA extracts also containing allelochemicals that can be used to regulate plant growth processes and create eco-friendly and resilient cities. The analysis of the stimulatory allelopathy index for 7 parameters demonstrates the direct effect of VA extracts in 62.3% of cases. The variability of the broad spectrum of effects of VA extracts of different host trees on the ontogenesis of *C. maxima* plants shows the presence of potential allelochemicals, resulting from the vital products of the host-parasite relationship. These effects are not fully explained by total polyphenol content and antioxidant activity as in previous studies of other mistletoe species. The authors consider this work a pilot study that expands the areas of application of VA extracts and knowledge about potential sources of allelochemicals.

## Introduction

European mistletoe (*Viscum album* L.) is an evergreen perennial aerial hemiparasitic plant with a special mode of cellular respiration that parasitizes various tree species and is distributed in Europe, Africa, America, Australia, and Asia^1,2^. Mistletoe spreads actively in anthropogenic ecosystems and in some cases reaches 80% faster spreading than in natural ecosystems^3,4^. Thus, it creates a problem for green urban areas (reduces plants’ decorative effect and vitality), which cannot be solved without damaging the host plant and when lacking control technologies. Our research is a pilot study of the effect of VA extracts from different hosts on plants, which could be a practical solution to utilizing VA in the city and have social and environmental impact to create a resilient city.

Most research papers are devoted to the medicinal properties of VA^5,6^, including anti-cancer^7–9^, antidiabetic^10^, cardiac activity^11,12^, anti-inflammatory^13^, immunomodulatory^14^, and antimicrobial^15^.

The mistletoe lectins and viscotoxins of VA aqueous extracts have been used in medicine for 80 years^16–18^. In addition, chemical compounds of different molecular sizes (from ≤ 1500 Da), such as phenolic acids, flavonoids, tannins, terpenoids, phytosterol, saponins and organic acids were identified in VA extracts^19,20^. Different antioxidant activities of mistletoe depending on the host plant, time of collection, plant organs and extracting solvent were found^21–24^. The combination of different host species and abiotic factors may result from the wide variability of mistletoe’s chemical composition and biological activity^21,22^. A detailed analysis of the available literature allowed the scientists to distribute in percentage expressions the chemical classes in VA where alkaloids and their derivatives > 40%, phenols and their derivatives > 20%, terpenoids and their derivatives > 10%, fatty acids and their derivatives < 10%, carbohydrates and their derivatives < 5%, other < 3%^23^. In addition, scientists have identified 212 metabolites in fermented aqueous extracts of VA from different host trees by MS and MS/MS analysis^20^. Numerous studies demonstrate the effectiveness of water extracts in medicine^24^, but the issue of the effect of water extracts from mistletoe on plants is poorly understood and still open.

The term “allopathy” was first formally coined by scientist Hans Molisch in 1937 and refined by Rice in 1984; defined as «a biochemical relationship between all plants, including microorganisms that can have a direct or indirect, harmful or beneficial effect of one plant on another through its produced chemical compounds in the environment»^25^. In 1996, the International Society of Allelopathy reinterpreted the definition of “allelopathy” by adding any process involving secondary metabolites produced by plants, fungi, microorganisms and viruses that affect the growth processes of agricultural and biological systems^26^. A new conceptual vision of the term “allelopathy” is offered by Wardle et al.^27^ who define it as a mechanism of interference that releases biochemical compounds that affect another plant. The ability of a species to survive and reproduce may be determined by its ability to release allelochemicals into the environment or even to tolerate the presence of allelochemicals released by other plants^28^. Even though allelopathy as a subdiscipline has been developing for a long time, it still has a history of a controversial nature due to the frequent equating of the allelopathic effect with the presence of a phytotoxic phytochemical substance and disregarding the water solubility of allelochemicals^29^.

For our experiment, we formed the following scientific questions and hypotheses:

### H01

It is based on the different effects of tested extracts on the experimental plants (EP) depending on the host tree and time of pre-treatment.

### H02

Allopathic properties of aqueous VA extracts can regulate the growth and development of the EP by dose–response relationship.

### A01

This assumption is based on the possibility of applying of VA aqueous extracts to plant growth, which will simultaneously solve part of the problem of mistletoe utilization in cities. The null hypothesis *H01* is based on the analysis of previous phytochemical screenings, which demonstrate high biological activity of VA plants by a wide variety of chemical compounds with different molecular sizes of primary and secondary metabolites. These show high probability of having an impact on the growth and development of the EP. VA extracts from different host plants are characterized by different phytochemical compositions, which means different effects on the same EP. The mistletoe-host system is unique from an eco-metabolic point of view because mistletoe derives its metabolites mainly from the primary metabolism of its host tree and synthesises its own defence compounds. Moreover, long-term parasitism of mistletoe generates a new metabolic identity of the host plant^30^.

Allelochemicals are defined as bioactive secondary metabolites with an unspecified function in primary metabolism in response to biotic and abiotic stress^31^. Generally, allelochemicals include various classes of chemical compounds, mainly phenolic compounds, terpenoids, alkaloids and nitrogen-containing chemicals, and many others^32^. Hypothesis H02 assumes the presence of high content of plant allelochemicals in VA aqueous extracts, such as phenols, that can affect the processes of germination, root growth, and photosynthesis^33^. Scientists note that different types of mistletoe demonstrate allelopathy activity^34,35^. In addition, the results of recent studies suggest the possibility of using the antioxidant activity of plants as an indicator for selecting plants for screening potential allopathic species^36^. Moreover, allelochemicals are well known to induce hormesis at high and low concentrations. To verify these aspects, we carried out short-term (2 h) and long-term (24 h) pre-treatment of seeds with test extracts^37^. Thus, the question for us in H02 was whether the biochemical compounds of the test extracts could have the properties of allelochemicals.

The truth or falsity of A01 will depend on the results of the previous hypotheses; it is only a conclusion. The use of biological products in urban landscaping to reduce the pesticide load on urban areas should become a priority to solve a set of problems with increasing plant viability and protecting them from the effects of urban environmental factors.

## Materials and methods

### Plant material

**Figure 1:**
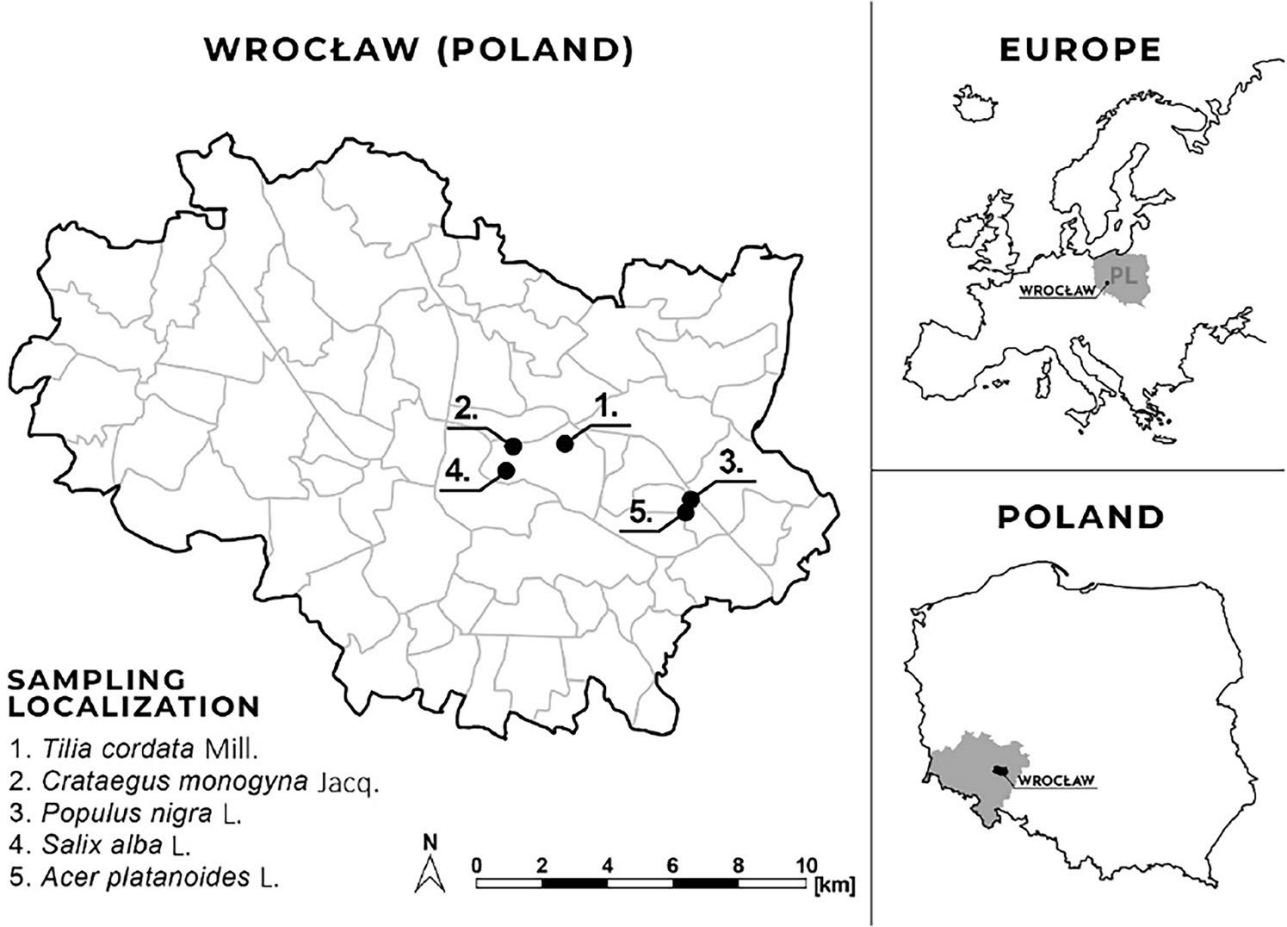
Geolocation of research *Viscum album* L. of different host tree species.

The samples of VA were collected in March 2023 during VA fruiting period from different parts of urban green spaces in Wroclaw by arborists of the Greenery Department of the city. All plants were taxonomically authenticated at the Wroclaw University of Environmental and Life Sciences (WUELS), Poland. The plant material used in the study was collected following current legislation and does not violate IUCN records. VA was collected from deciduous trees by a professional arborist with permission from the Management of Urban Greenery in Wroclaw. Five dominant different host tree species were selected for the study, namely, *Tilia cordata* Mill., *Glog monogyna* Jacq., *Populus nigra* L., *Salix alba* L. and *Acer platonoides* L.^38^. We selected 4 urban trees of the same species with the same intensity of mistletoe infestation (10–30 bushes per tree) in one location (Fig. 1)^39^. From each tree, 3 bushes (a whole mistletoe plant) were collected at different heights and combined into one experimental sample. The plants were then stored in dark plastic bags, separated into leaves, shoots and berries, and dried.

The unsteamed seeds of *Cucurbita maxima* ‘Hokkaido orange’ were purchased from a local gardening store in March 2023. It was a certified seed material with high germination capacity. The results of our measurements (n = 10) demonstrate the following seed characteristics:100 seeds’ mass 20,5 ± 1,22 g, length 13,8 ± 2,32 mm, width 1,2 ± 0,30 mm. We used AXIS D1000 laboratory balance to measure the weight, and INSIZE calliper rod to measure the length and width (see Supplementary Fig. S1 online).

### Preparation of aqueous extracts and seed treatment

The shoots with leaves were washed in the tap water in the laboratory and the remaining water was removed in MPW-350R centrifuge^40^. We put the plant material in an oven at 50 °C for 3 days and packed it in black plastic bags until use. The dried plant material was placed in a blender for 90 s and mixed with a spatula every 30 s. To 10 g of dry crushed mistletoe was added 100 ml of sterile distilled water and centrifuged twice for 30 min at 3220 g (20 °C) and 20,817 g (room temperature)^41^ (see Supplementary Fig. S2 online). The plant extract was filtered through filter paper and then stored in a refrigerator^42^.

We pre-treated pumpkin by covering the seeds with 50 ml of 5 VA extracts and distilled water for 2 h (Test A) and 24 h (Test B), stirring periodically at room temperature in the dark. After that, 20 seeds were placed in a sterilised petri dish (18 cm) on filter paper on both sides in triplicate (Table 1).

**Table 1:** Scheme of the experiment on germination of pumpkin seeds with pre-germination treatment with aqueous VA extracts and treatment time.

No	Host-tree	Seeds (n = 3)	Time pre-sowing seed treatment	
1	*Tilia cordata* Mill.	20	Test A 2 h	Test B 24 h
2	*Crateagus monogyna* Jacq.			
3	*Populus nigra* L.			
4	*Salix alba* L.			
5	*Acer platonoides* L.			
K	distilled water			

The dishes were placed in ST 1450 CS SMART thermostat at ± 24 °C with constant light (see Supplementary Fig. S3 online). Only seeds with at least 2-mm-long radicles were included in calculations^33,34^. We did not add water to the seeds for the first two days as the filter paper was moistened. On the following days of observation, we added 20 ml of distilled water to each dish daily. Thus, we obtained 36 Petri dishes for the experiment and observed 720 seeds.

### Effect of VA extracts on plants

#### Seed germination rates (SRG)

To study the effect of mistletoe aqueous extracts on seed germination, the following indicators were calculated and analysed: germination percentage (GP, %), germination index (GI), last day of germination (LDG), time spread of germination (TSG), mean germination time (MGT), and seedling vigour index (SVI). The following formulas were used to calculate the seed values:

GP = ∑ seeds germinated per day/∑ seeds placed for germination × 100^43^.

GI = ∑G1/GD1 + ∑G2/GD2 + ··· + ∑Gx/GDx, where G—number of germinated seeds, GD—germination day^44^.

LDG = last day of seed germination^45^.

TSG = LDG—day on which the first germination event occurred^46^.

MGT = ∑ (ni × di)/N, where ni—number of seeds germinated a day, N—total number of germinated seeds at the end of experiment^44^.

SVI = (Mean root length + Mean shoot length) × % germination^40,47^.

#### Inhibitory and allopathic effects

We analysed the morphometric parameters of the seeds every 24 h during the 8 days of laboratory research. Measurements were made using an INSIZE calliper with an accuracy of 0.01 mm. In general, we made 5760 observations. The extracts’ inhibition percentage index (IP) was calculated using the formula, where IP < 0 represents an inhibitory effect, and IP > 0 represents a promoting effect.

IP = [1 − (LE/LC)] × 100^48^,

LE = length of seedlings in aqueous plant extract.

LC = length of seedlings in control (without extract).

We calculated the allopathic effect of the extracts by the stimulatory allelopathy index (SAI): SAI = (T/C − 1) × 100, where T—treatment value and C—control value^49^. We estimated the SAI for GP and SVI indicators of the last day of germination in dishes (8 GD), water content (WC), photosynthetic pigments and TPC^50^.

We grouped the results of the SAI indices using a 5-point scale, where negative SAI values were marked in red and positive values in green (Table 2).

**Table 2:** Description of the SAI impact scale with 5 categories.

Score	SAI, %		Effect
0	− 10–0	0–10	Very low
1	− 11–(− 30)	11–30	Low
2	− 31–(− 60)	31–60	Moderate
3	− 61–(− 100)	61–100	High
4	> − 100	> 100	Very high

#### Chlorophyll and carotenoids content

After the SRG assessment, we planted the all experimental plants and even seeds (2B) that did not germinate in pallet containers (65 × 60 × 48 mm, 190 cm^3^) in peat TM PlanzSubstrat “Athena Mieszanka torfowa Substrat” (see Supplementary Fig. S4 online). Plants in pallets were placed in a thermostat ST 1450 CS SMART at a temperature of ± 24 °C with constant light. Every day we moistened the soil with distilled water and observed the plants. The aboveground part of the experimental plants was cut off on 20th day (Test A) and 21th day (Test B) after the beginning of the bio-test. To determine the photosynthetic pigments, we weighed 400 mg of fresh leaf weight of the experimental plants on AXIS AD1000 balance with an accuracy of ± 0.01 (see Section “Chlorophyll and carotenoids content”). Chlorophyll *a*, *b*, and carotenoids were extracted in a clean mortar by grinding fresh leaves of the experimental samples (400 mg) with sand and CaCO_3_. The acetone (80%) was added to the homogenized material and filtered using a water pump and a glass funnel. We determined the content of chlorophyll a and b, and carotenoids by the spectrophotometric method by Spectroquant Pharo100 MERCK^51^.

The content of photosynthetic pigments was calculated using the following formulas:

$${\text{Chl}}_{{\text{b}}} = \frac{22,9 A645 - 4,68A633}{{20*m}} \;\left[ {{\text{mg g}}^{{ - {1}}} } \right]$$

$${\text{Car }} = \frac{1000A470 - 3,27Chla - 104Chlb}{{229}}\;\left[ {{\text{mg g}}^{{ - {1}}} } \right]$$

where m—weight of fresh leaves.

#### Total phenolic content (TPC)

TPC was determined in ethanolic extracts by the Folin-Ciocalteu method in terms of the Gallic acid equivalent (GAE) in mg/g of the extract^52,53^. We mixed 100 μl plant extract with 2000 μl distilled water, 200 μl Folin-Ciocalteu reagent and 100 μl of 15% Na_2_CO_3_ and stored it in darkness for 2 h. The absorbance was recorded at 765 nm by Spectroquant Pharo100 MERCK. All measurements were performed in three replications^54^.

#### Water content and plant biomass

The experimental plants growing in pallets were cut off and weighed on AXIS AD1000 balance with an accuracy of ± 0.01 g to obtain fresh mass (FM). The samples were then placed in an oven and dried at 105 °C to a constant weight (DM). Calculations were performed using the following formula:

WC (%) = 100 − [(DM × 100)/FM]^55^.

### VA extracts antioxidant activity

#### DPPH

The free-radical scavenging activity of the VA extracts was carried out by using the 2,2-diphenyl-1-picrylhydrazyl method with some modifications^56^. We prepared samples for the experiment by adding 500 μl of plant extract to 1.5 ml of ethanol and 0.5 ml DPPH. Then, we prepared a blank sample by adding 500 μl of distilled water to 1.5 ml of ethanol and 0.5 ml of DPPH solution. The absorbance was measured at 517 nm using Spectroquant Pharo100 MERCK. The results were calculated using the formulas and expressed as mg Trolox (TE) per 100 g dry extract.

#### FRAP

FRAP assay was followed according to the method proposed by Benzie and Strain^57^ with some modification. The 300 mM sodium acetate (CH_3_COONa) buffer solution at pH 3.6, 10 mM TPTZ (2, 4, 6-tripyridyl-s-triazine) solution, and 20 mM ferric chloride (FeCl3) solution were mixed in the ratio of 10:1:1^58^. The plant extracts were mixed with 1.9 mL FRAP reagent and the absorbance was measured at 593 nm after 10 min using Spectroquant Pharo100 MERCK. The values were expressed in dry weight of the plant samples as μM of ferrous equivalent Fe (II) per gram of sample.

### Statistical analysis

All obtained experimental data was analysed using Microsoft Excel, JupyterLab (Python 3.9), and RStudio (R 4.3)^59^. Statistical similarity, according to the GD indicator, between seed samples treated with mistletoe aqueous extracts from different host trees and distilled water (control) was assessed by comparing 95% confidence intervals^60^. These intervals were calculated based on the binomial distribution of proportions. For other indicators, similar statistical analyses were conducted using classical parametric (LDG, TSG, MGT, GI, as well as TPC, DPPH and FRAP contents) and nonparametric (box plots for TPC, DPPH and FRAP contents) criteria. Heat maps with hierarchical clustering were created using the Heatmapper web platform, employing the Pearson distance measurement method^61^.

## Results

### Germination bioassay

**Figure 2:**
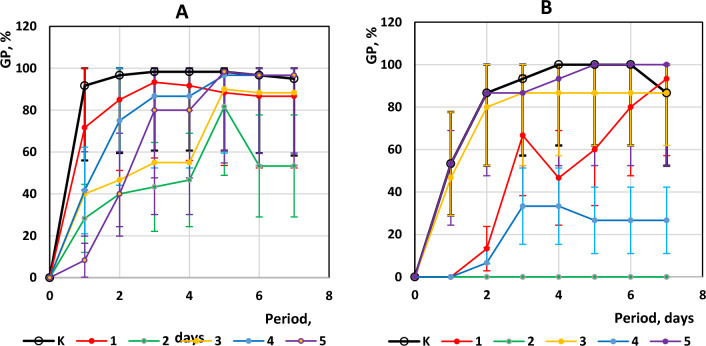
GP of *Cucurbita maxima* ‘Hokkaido orange’ with 2 h pre-sowing treatment (**A**) and 24 h pre-sowing treatment (**B**) using mistletoe aqueous extracts from different hosts-tree: error bars are confidence intervals at 95% probability.

The analysis of seed germination rates of seeds treated with VA extracts shows different indicators on different days of the experiment for test samples, which indicates the direct influence of the components of the aqueous extracts and the duration of pre-sowing seed treatment on the growth and development of plants. The confidence intervals indicate statistically significant differences in the test results for certain samples, underscoring the importance of the research factor (see Fig. 2). For seeds with 2-h treatment (Test A), the highest GP indices were recorded for KA, 1A and 4A and were 98.3%. Not significantly lower indices of 96.7% were recorded for 5A and 3A. Our observations recorded a decrease in GP indices in the last 3 days of the experiment due to the loss of seed vitality in some research samples, except for seeds of 3A and 5A, where the indices were stable. This phytotoxic effect of the extract was most expressed in extract 2A from *C. monogyna*, where more than 30% of seeds lost their vitality within a day.

In spite of the results of Test A, where 2A demonstrated a decrease in GP indices up to 40%, Test B reliably indicates the phytotoxic effect of mistletoe extract from the *C. monogyna* (2B, GP = 0%) and partially 4B extract from *S. alba* (GP = 26.6%). The germinated seeds treated with 4B extract had the highest morphometric parameters among all EP, which suggests the phytotoxicity of this extract and, at the same time, allopathic properties. In addition, on the 4th day of the experiment, some seeds in test 1B began to lose the vitality of the first formed root, and at the same time, the seeds did not show any morphometric changes for 2 days in VA-*T. cordata* extract with the highest DPPH values (44,69 ± 2.11 TE/g) among all test extracts. However, on the 6 GD, newly branched roots and leaves began to form actively, so we can observe such dynamic changes in the indicators on the graph, which we will try to explain in the Discussion section.

The shorter time treatment by VA extracts of the experimental seeds inhibited the speed of germination rates (GR) compared to the control (2–3 days). The test seeds treated with the VA extract from *C. monogyna* did not germinate at all on the 8th GD of the experiment. It was found that long-term treatment with the extracts did not significantly affect the rapidity of GR of seeds of samples 4B and 5B compared to 4A and 5A. However, the extract of 3B stimulated GR, and 1B, and, on the contrary, inhibited it compared to KB.

**Figure 3:**
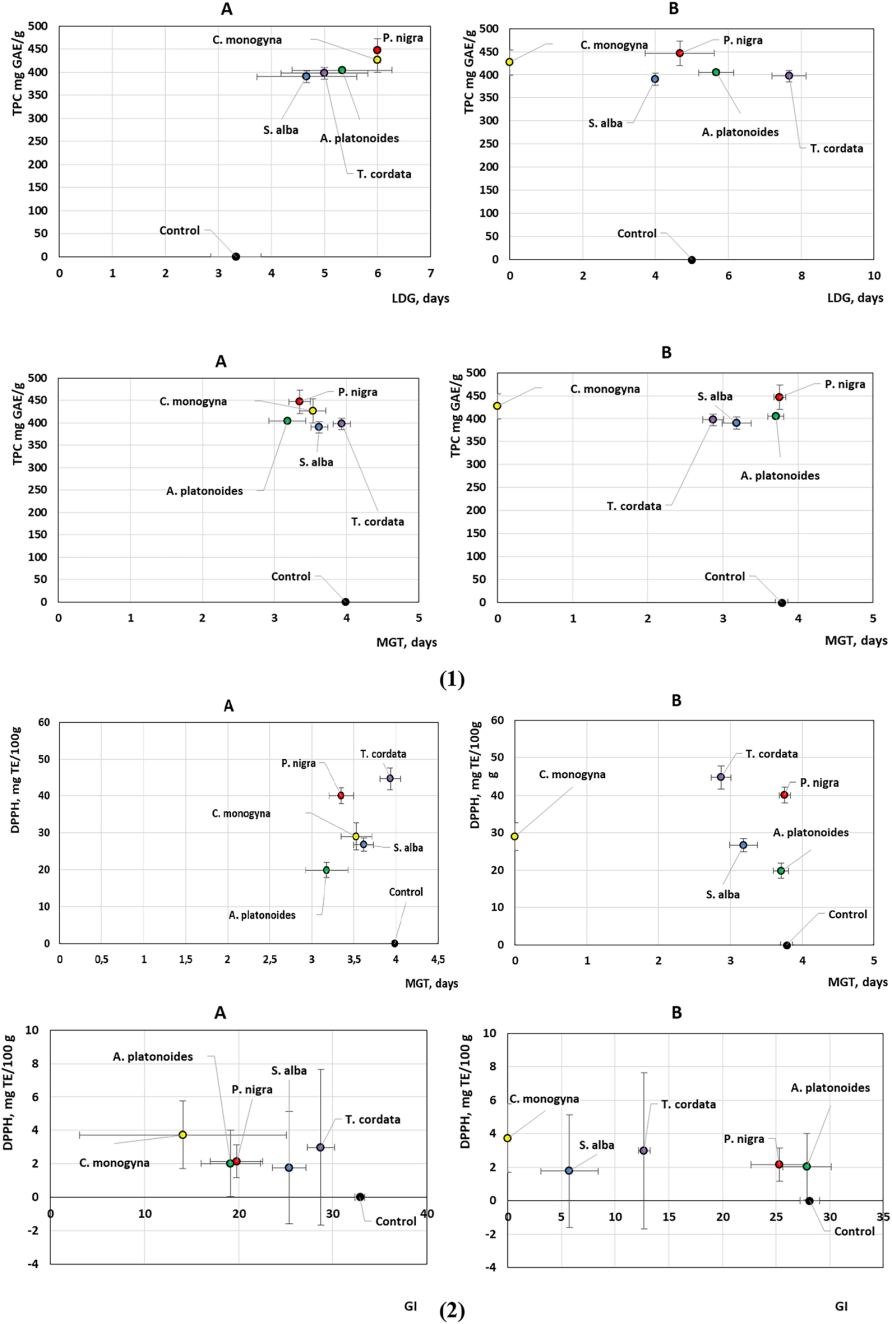

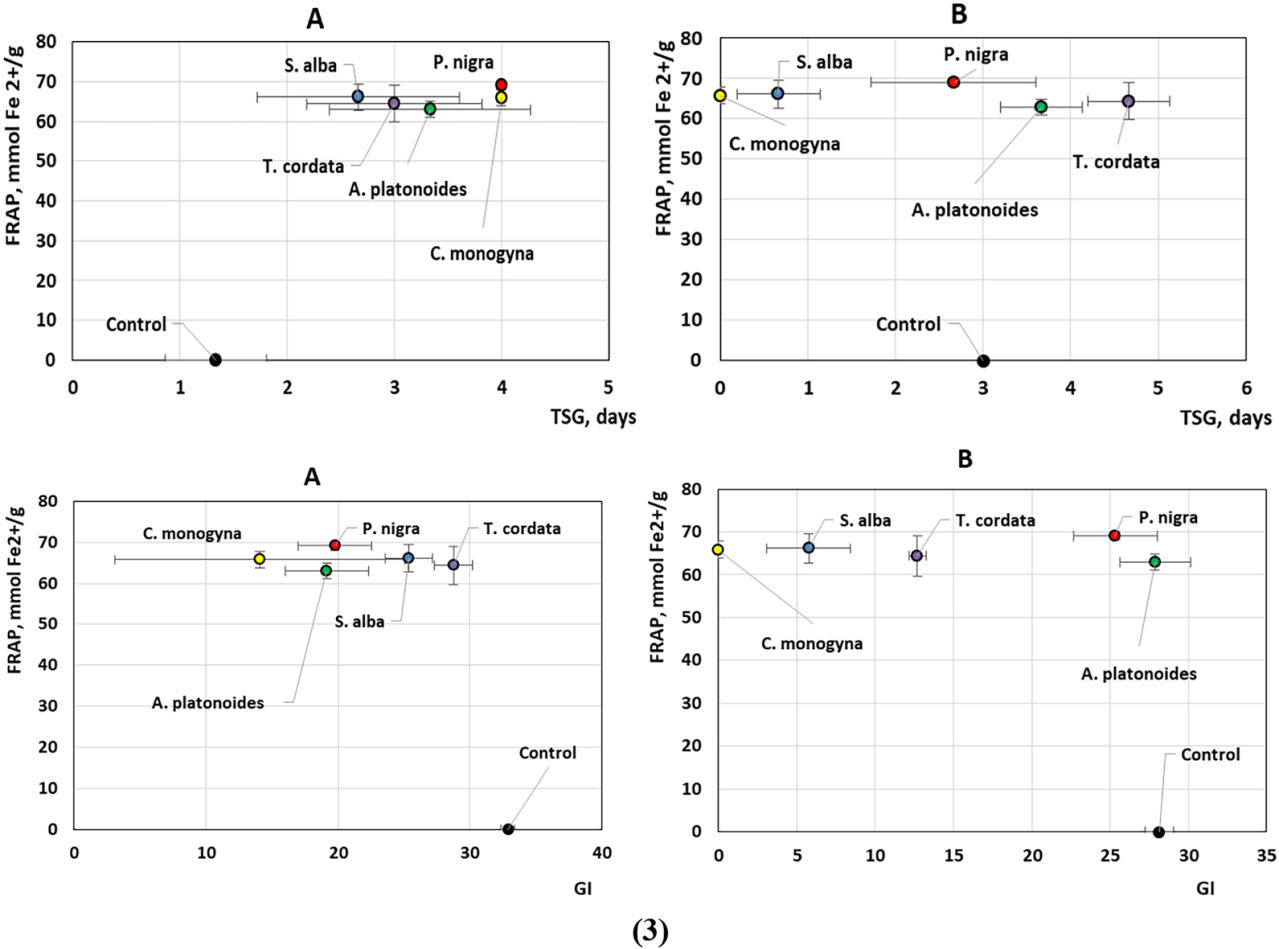
Dependence of TPC contents (1) and antioxidant activity (DPPH (2) and FRAP (3)) in mistletoe aqueous extracts, obtained from different host trees, on the germination speed of *Cucurbita maxima *‘Hokkaido Orange’ test seeds treated for 2 h (**A**) and 24 h (**B**) before sowing.

The analysis of the graphs of the main effects of the components of the experimental VA extracts on pumpkin seeds shows different response rates in SRG depending on the content of phenolic compounds, and antioxidant properties of the extracts depending on the host tree (Fig. 3 and see Supplementary Fig. S3 online). We have recorded the signal of response in Test A with short-term seed treatment in LDG, TSG and GI, depending on the TPC in the test VA extracts. Simultaneously, the concentrations of all the aforementioned substances exhibit a strong correlation with each other, with correlation coefficients (r) greater than 0.82. We also detected an effect on MGT and GI values of DPPH in Test A and a slightly weaker signal on LDG and TSG values (FRAP).

In contrast to Test A, the results of the analysis did not reveal a significant effect of phenolic compounds in Test B with long-term treatment of VA extracts on the germination rate of experimental seeds. A weak influence of FRAP on TSG was recorded.

**Figure 4:**
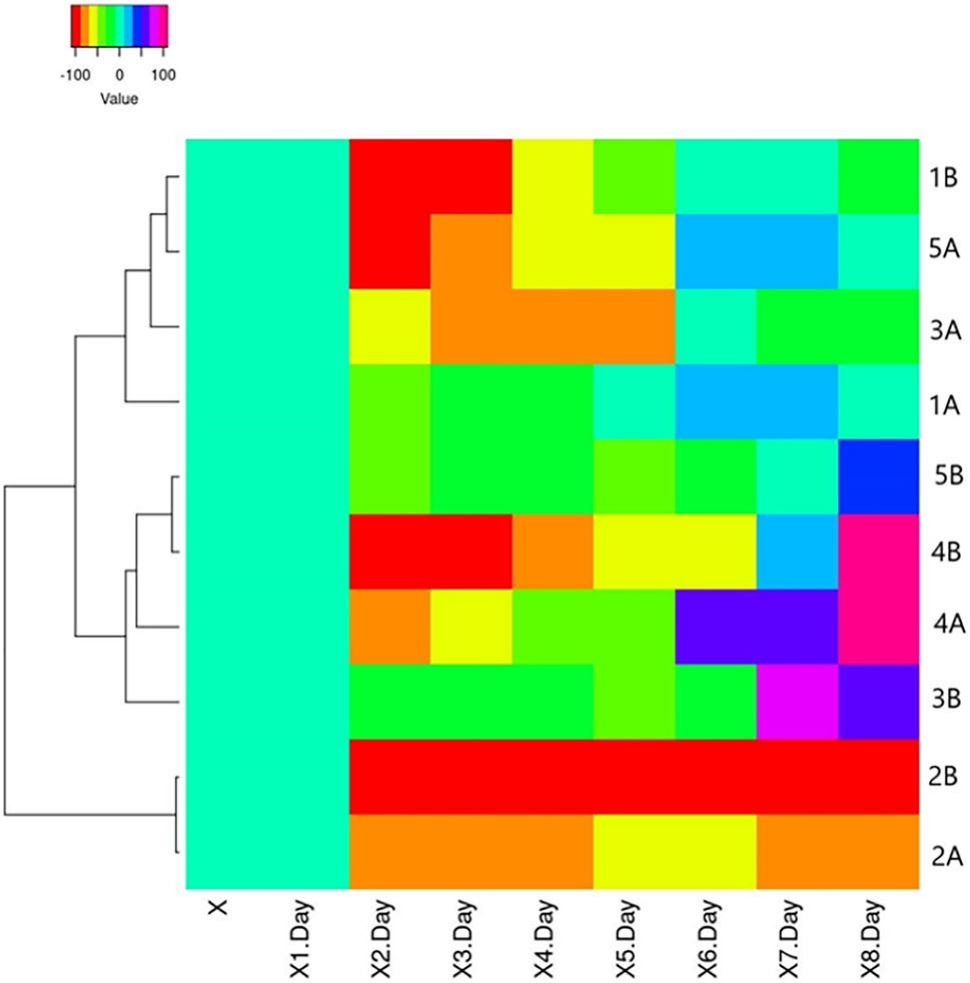
Heat map representation of SVI of *Cucurbita maxima* ‘Hokkaido orange’ seeds with priming treatment by mistletoe extracts compared to control (Test A—KA) and (Test B—KB), (  < 0.05).

The daily analysis of the SVI index shows lower indicators of all research plants up to 4 days of the experiment regardless of the duration of pre-sowing seed treatment compared to the control. For the first time, the dominance of SVI indexes of treated seeds with extracts over KA was recorded in Test A at 5 GD of trial 1A (+ 4%) and 6 GD of trial 1A (+ 26%) and 4 A (+ 52%). Thus, in Test B, on GD 7, higher values were recorded for trials 3B (+ 71%) and 4B (+ 12%) compared to KB. On the 8th day, the highest values were recorded for plants that can be arranged in descending order 4A > 4B > 3B > 5B compared to the control in their bio-test KA or KB (Fig. 4).

### Evaluation of the effect of extracts on growth processes

#### Inhibitory effect

**Figure 5:**
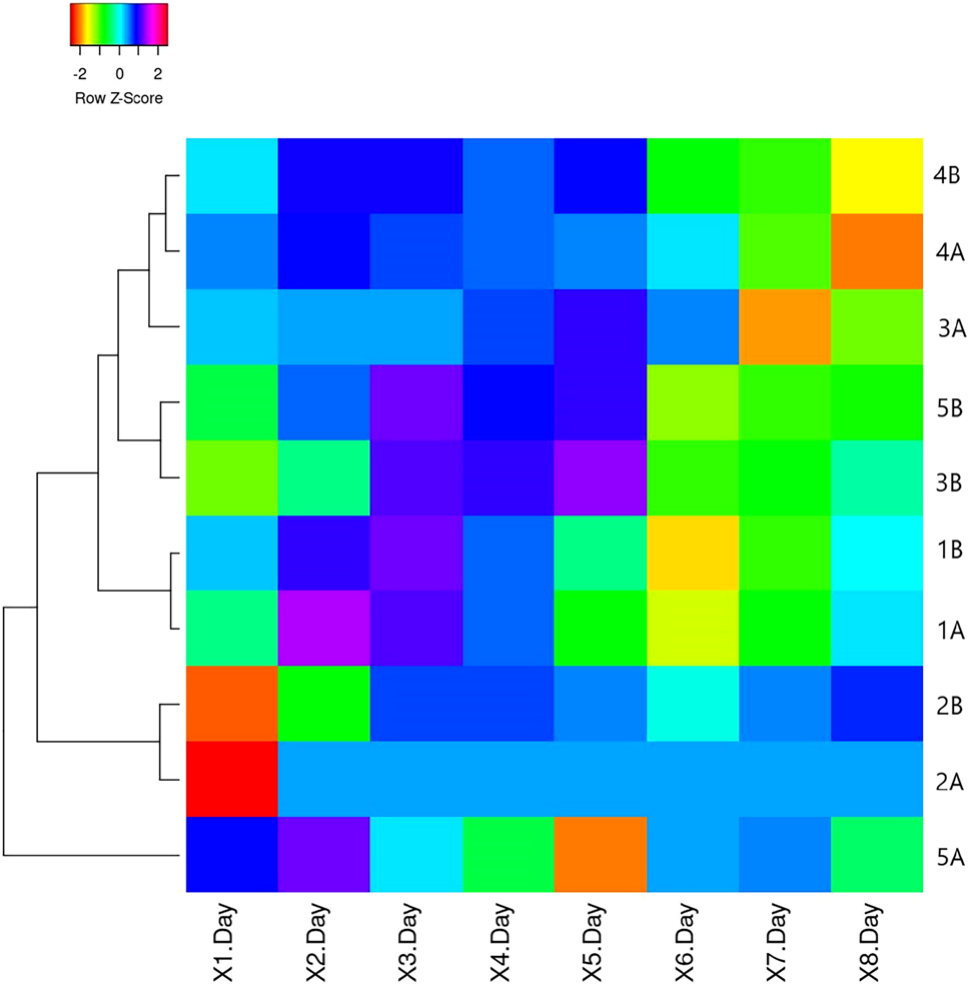
Heat map representation of annual IP (8 days) of *Cucurbita pepo* ‘Hokkaido orange’ seeds with priming treatment by mistletoe extracts, (  < 0.05).

Results of the daily IP analysis indicate variable activity of this indicator on different days of the experiment (Fig. 5). By the first 4–5 GD of all samples had IP > 0 except for test 5B, which had IP < 0 from day 3rd GD. We found that test 2B had a 100% inhibitory effect because no seed germinated in 8 days. Thus, on the last day of the experiment in dishes (8 GD), negative IP values in Test A were fixed for 1A, 4A, 5A and in Test B for 3B, 4B, 5B.

#### Allopathic effects

The results of the experiment indicate the effect of VA aqueous extracts on the growth and development of experimental plants at different levels of ontogeny (seed germination, morphometry, physiological levels) (Table 3). The duration of pre-sowing treatment significantly affects (Test B) the SAI values of various indices with a wide variation. In some tests, SAI results show a change from negative to positive values (GP 1A-1B, SVI 3A-3B, LA 4A-4B, 5A-5B, chl*a* and car 2A-2B, 4A-4B), which indicates the possibility of changing inhibition to stimulation depending on the treatment duration.

**Table 3:**
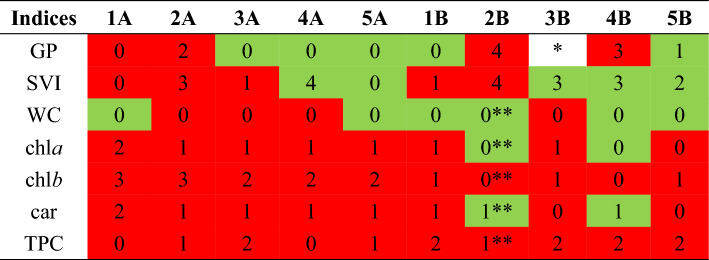
Scale indices SAI of *Cucurbita pepo* ‘Hokkaido orange’.

Red colour—negative effect, green colour—positive effect

*No effect, ** studies were conducted on one plant in triplicate

It was experimentally established that 37.7% of the results obtained for assessing the effect of VA extracts on 7 parameters of experimental plants had a negligible effect on plants and were evaluated as 0 points (Very Low). In addition, the data indicate the presence of the effect of extracts on different levels of plant organization, where 31.9% were evaluated as Low, 17.4%—Moderate, 8.7%—Very High and 4.3%—High.

### Phenolic content and antioxidant activity of VA extracts

**Figure 6:**
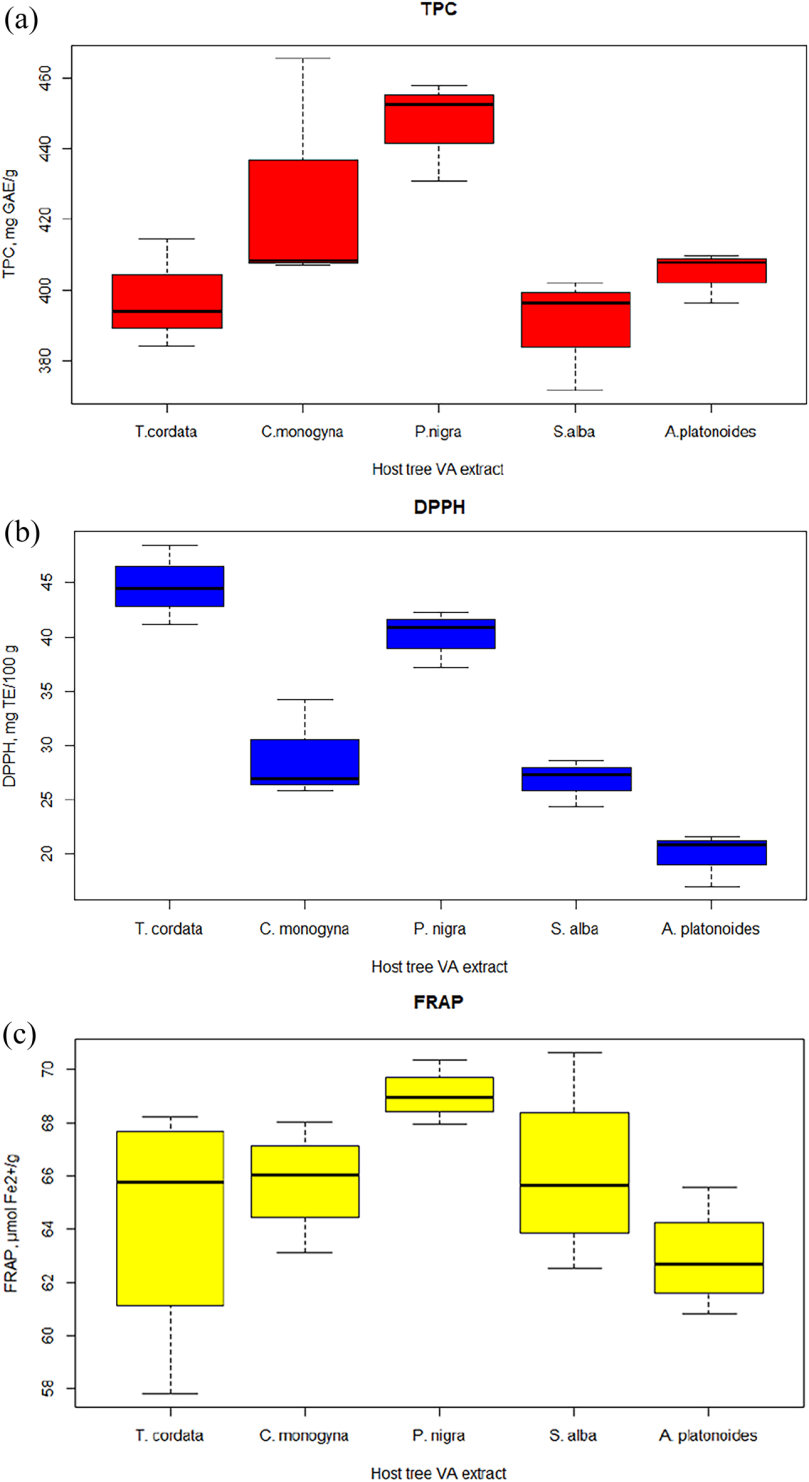
Box plots of TPC (**a**) and antioxidant activity (**b**, **c**) of aqueous extracts of *Viscum album* L. from different host trees.

Different content of phenolic compounds in the experimental VA extracts was found depending on the host tree. The values ranged from 390.01 ± 9.4 (VA-*S. alba*) to 446.9 ± 8.3 mg GAE/g (VA-*P. nigra*) (Fig. 6). In addition, the antioxidant activity of VA extracts in terms of DPPH and FRAP differed from each other. Thus, according to DPPH, the extracts can be arranged in the following sequence: *T. cordata* > *P. nigra* > *C. monogyna* > *S. alba* > *A. platonoides* and FRAP *P. nigra* > *S. alba* > *C. monogyna* > *T. cordata* > *A. platonoides*.

## Discussion

Scientists noted germination rate and germination indices are the most sensitive to allelochemical relationships but the most appropriate is to compare experimental samples with controls^62,63^. The EP germination rate (LDG) in Test A is correlated with the TPC of VA extracts, where, for example, seeds treated with VA-*P. nigra* had the highest LDG, and the extract had the highest TPC, but in Test B this trend is absent. A similar phytotoxic effect of extracts from *Triticum aestivum* L. was recorded for GP and germination time of *Portulaca oleracea* L. and *Stellaria.* *Media* (L.) Vill., which is explained by the high content of TPC and flavonoids^64^. A similar allopathic inhibition effect of sesame extracts on seed germination of *Phyllostachys edulis* (Carri-ere) J. Houz was found^65^. It was established that phenolic compounds from *Festulolium* genotypes have the greatest allelopathic effect on the growth processes of *Eruca sativa* L. (Rocket)^66^. The duration of treatment with VA extracts significantly affects the growth and development of plants, which may be due to the different effects of water-soluble and non-water-soluble metabolites of extracts, including water-soluble phenolic compounds (WSPC). Scientists assume that the most effective allelochemical compounds are characterized by limited solubility in water, so it is very important to consider their water solubility^29^.

On 4GD of the experiment, EP in sample 1B began to lose their vigour due to the death of the first formed main root, but after 2 days, the root system began to actively form from lateral roots. In *C. peppo*, lateral root initiation (LRI) and development occur during embryogenesis and directly in the apical meristem of the parental root, followed by rapid monopodial branching of the main root^67^. In addition, in the radicle of *Cucurbitaceae*, during embryogenesis, the first 3–5 LRI can be formed^68^. Notably, we recorded the highest DPPH content in this VA extract (1B), which could presumably affect root formation processes, but this is only an assumption as to why we could observe such results in Test A. The results of studies of 55 plant species show that antioxidant activity, including DPPH, demonstrates inhibitory properties and is considered by scientists as a potential allopathic source^36^.

Allelochemicals are most often represented by secondary metabolites in plant cells, which are produced in response to external factors, but have no recognized specific function in the plant^33^. It was determined that WSPCs play the role of endogenous inhibitors in the seed coat of *Triticum aestivum* L. seeds to control the the germination process, which is partially caused by the inhibition of peroxidase reactivation^69^. The results of our experiments demonstrate the lowest germination rates (GP) for long-term treatment (Test B) of seeds with extracts of pre-treatment of seeds with VA- *C. monogyna* (GP = 0%) and VA-*S.alba* (GP = 27%). This indicates inhibition in contrast to the short-term pre-treatment (Test A) from the long-term pre-treatment (Test B). The phytotoxic effect of mistletoe extractsof *Dendrophthoe falcata* (L.F.) Ettingsh. on the germination of seeds of redhead and green millet was determined, which is the result of metabolic disorders due to the influence of allelochemicals^34^.

Our research confirms the findings of previous scientists about the positive effect of mistletoe extracts in some cases, where some of the VA test extracts showed high GP values similar to the control and only in one case slightly higher than the control (Test B, VA-*A. platonoides*). It was found that the seeds of *Pennisetum glaucum* [L.] R. Br. treated with aqueous VA extracts had higher germination rate and germination energy compared to the control, increased the resistance of experimental plants to pearl millet downy mildew pathogen probably due to the presence of amides and other aromatic compounds, and increased the activity of peroxidase and phenylalanine ammonia kinase in experimental seedlings^40^. Presumably, the different reaction of *C. maxima* ‘Hokkaido orange’ seeds to the duration of pre-sowing treatment with VA extracts of different host trees is caused by different sensitivity of seeds to allopathic chemical compounds of different molecular sizes that are modified in water and the degree of penetration through the seed coat during long-term 24-h treatment.

The data indicate the inhibitory effect of aqueous VA-*T. cordata* and VA-*C. monogyna* extracts on the morphometric parameters of *C. maxima* ‘Hokkaido orange’ regardless of the duration of pre-treatment of EP. Different time of pre-treatment of *Lactuca sativa* L. seeds with aqueous extracts of *Achnatherum splendens* (Trin.) Nevski, *Artemisia frigida* Willd. and *Stella chamaejasme* L. did not reveal significant differences in the effect on lettuce seed germination. Still, all test extracts that inhibited lettuce seed germination and root length contributed to the increase in lettuce shoot length, stem length, leaf length and leaf width^70^. Scientists note that allelochemicals from plants are chemically diverse and include various phenolic compounds, terpenoids, alkaloids, and nitrogen-containing chemicals and many other chemical families^32,69^. We found a stimulating effect of VA-*P. nigra* and VA-*A. platonoides* in Test B, VA-*S. alba* in both tests. Based on the example of permeability of seed coats of *Allium cepa* L., *Phaseolus vulgaris* L., *Capsicum annuum* L., *Lactuca sativa* L., *Solanum lycopersicum* L., and *Cucumis sativus* L., it was found that 10 compounds of different chemical classes that failed to penetrate the seed coat during seed impregnation were absorbed by roots or hypocotyls after visible germination^71^. The most vulnerable among the photosynthetic pigments to the influence of allelochemicals of VA extracts is chlr *b* in Test A, which was lower than in the control by more than 2 times. It was found that VA significantly affected the content of chlr *b* in 3 host species (apricot, almond and plum), while the effect on the content of chlr *a* and carotenoids was minimal^72^. It was established that aqueous extracts from the leaves of *Mentha* × *piperita* L. act as inhibitors or stimulators on the functioning of photosystem II and the content of chlorophylls in *Helianthus annuus* L. plants^73^.

## Conclusions

EP with 24 h pre-treatment with aqueous VA extracts have lower rates of seed germination but higher morphometric parameters compared to plants with 2 h pre-treatment. During the long-term treatment of *C. maxima* seeds with VA-*S. alba* and VA-*A. platonoides* (“host-tree metabolites vs. mistletoe + water”), a positive effect of the components was recorded. The highest indicators of TPC were recorded in the VA extracts of *Populus nigra* L. (446.9 ± 8.3 mg GAE/g), DPPH of *Tilia cordata* Mill. (44.68 ± 2.11 TE/g) and FRAP of *Populus nigra* L. (69.08 ± 0.703 mg TE/g). The most sensitive among photosynthetic pigments to the effect of VA extracts is chlr *a* in the short-term treatment of plants in Test A. The possibility of regulating the growth processes (inhibition and stimulation) of plants exemplified by *C. maxima* ‘Hokkaido orange’ by applying aqueous VA extracts was experimentally established. The experimental data confirm our H01 and partially H02, the effect of allelochemicals on *C. maxima* ‘Hokkaido orange’ was recorded, but its nature opens up new horizons for further scientific research. The experiment shows the presence of water-soluble metabolites in VA aqueous extracts that have a positive effect on the growth and development of *C. maxima* (stimulating effect). Still most cases of the experiment of another host tree demonstrate the inhibitory effect of the extract components. Of course, many questions remain unanswered after our study, but the fact that VA metabolites have an effect on the ontogenesis of *C. maxima* ‘Hokkaido orange’ plants that are highly biologically active is undeniable. Despite the presence of some correlations between the TPC content and the antioxidant activity of VA extracts with the ontogeny of the experimental plants, the authors do not exclude the influence of other biologically active substances of VA extracts or their synergy, which still needs to be clarified. This is a pilot study that should serve as a basis and/or a hint for the future research of the biological activity of mistletoe extracts. In our opinion, the solution to A01 is promising for creating environmentally friendly solutions for the resilient city and solving the problem of mistletoe utilization in urban areas. In addition, the variable quantitative and qualitative composition of mistletoe extracts depending on the host plants, which is the result of mistletoe vital activity, prompts the question and future scientific discussion whether these biochemical compounds can be considered allelochemicals.

### Supplementary Information

Supplementary Figures.
