# Supplementary material for: Pre-treatment of Cucurbita maxima ‘Hokkaido orange’ by Viscum album aqueous extracts in search of allelopathic potential
Source: Sci Rep. 2024 Jun 28;14:14927. doi: 10.1038/s41598-024-65918-0 (PMC11213859; doi:10.1038/s41598-024-65918-0)
Supplement: Supplementary file 1 — Supplementary Figures. [file 41598_2024_65918_MOESM1_ESM.docx]

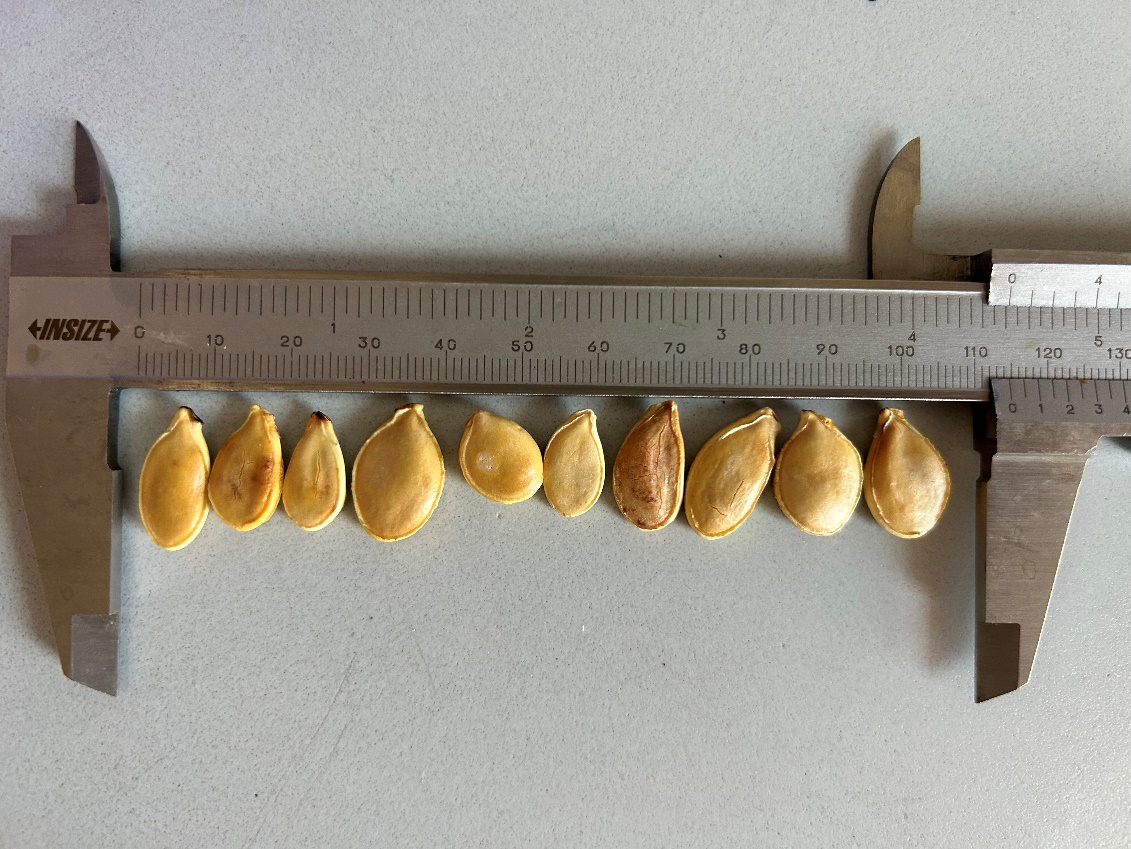
**Figure S1.** Experimental seeds of *Cucurbita maxima* ‘Hokkaido orange’.


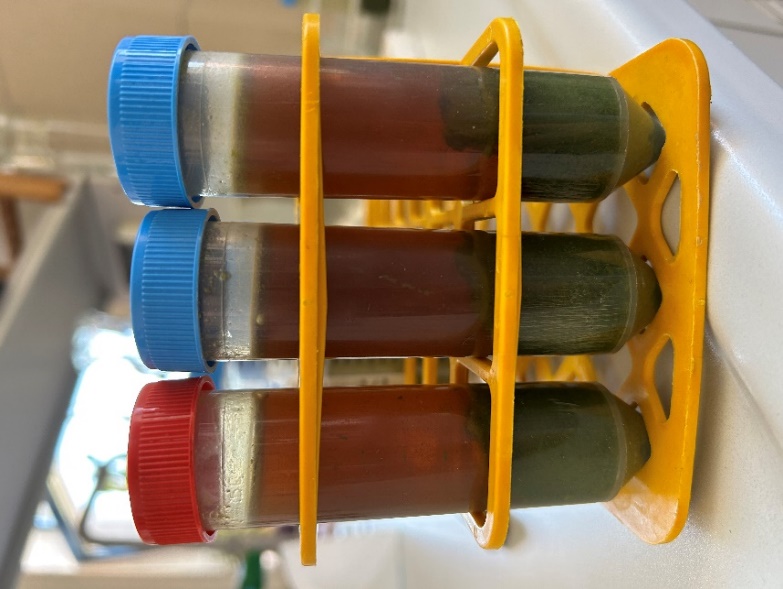


**Figure S2.** Mistletoe aqueous extracts after centrifugation.


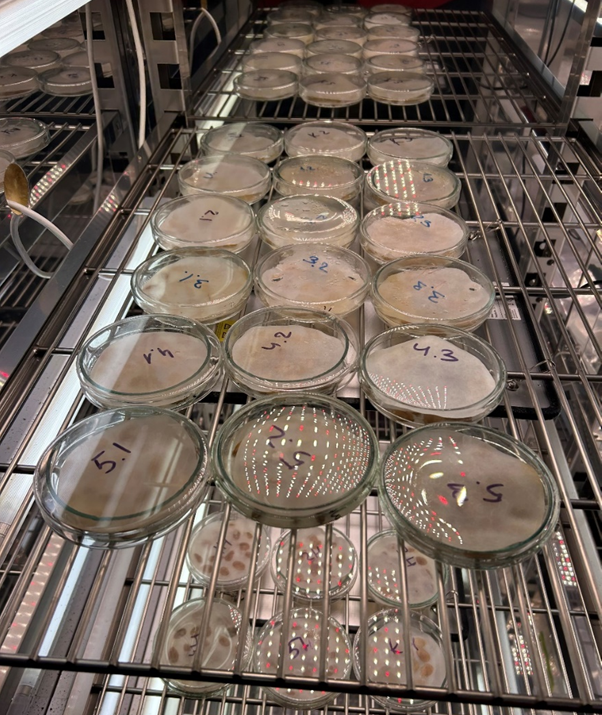


**Figure S3.** Germination test of seeds *Cucurbita maxima* ‘Hokkaido orange’.


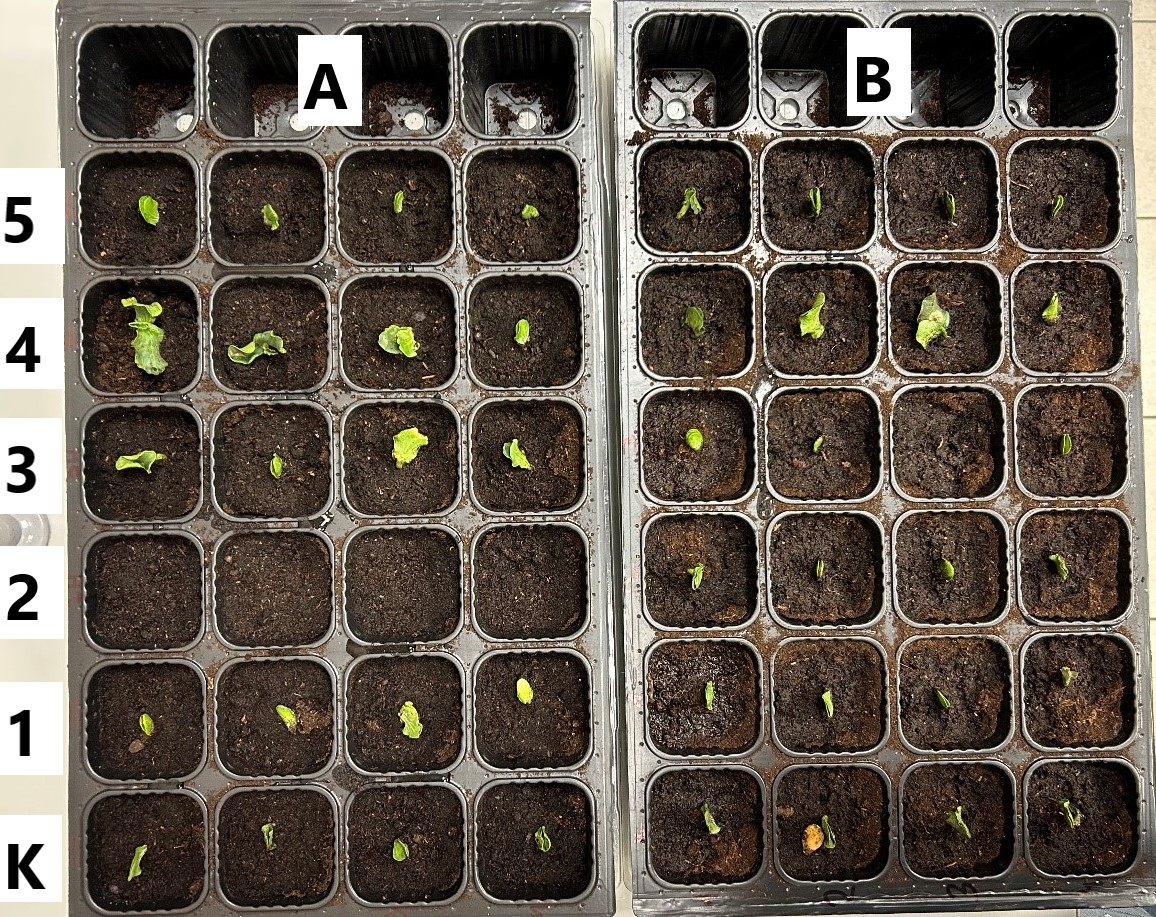


**Figure S4.** Experimental plants with pre-sowing treatment with different mistletoe aqueous extracts, day 11 (KA, 1A-5A) and day 10 (KB, 1B-5B).


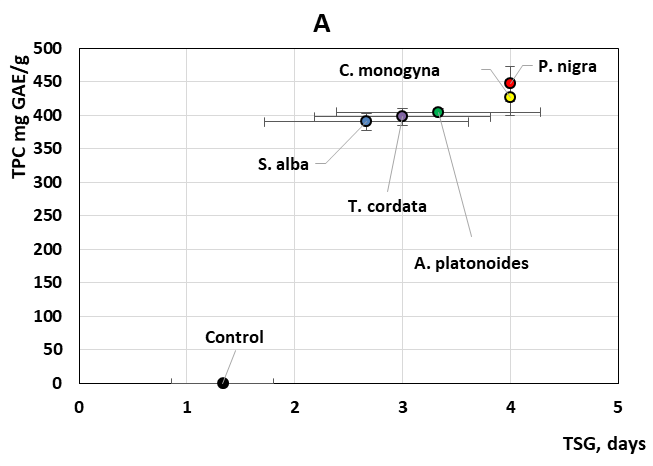

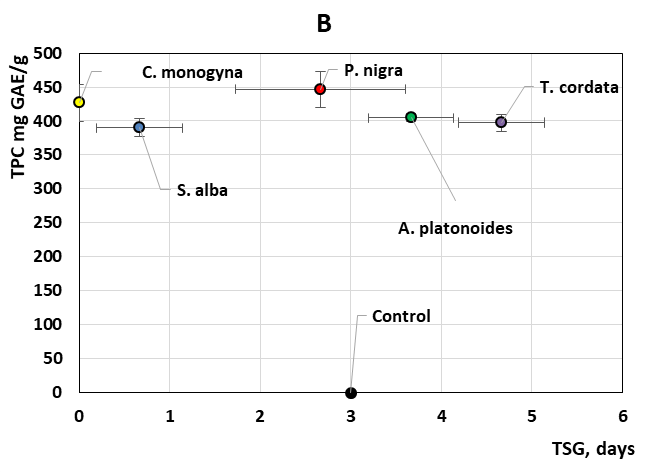


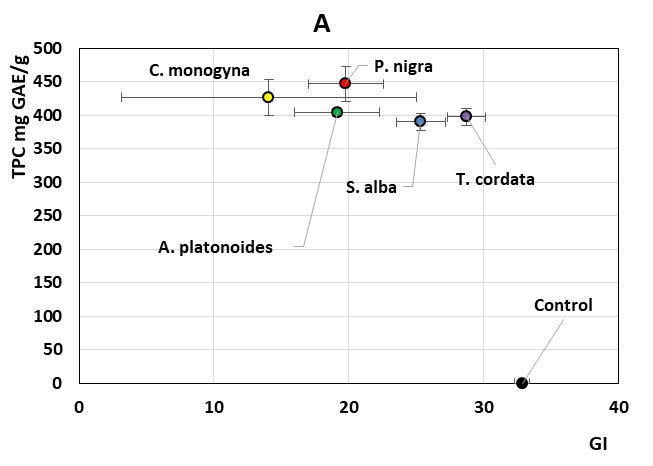

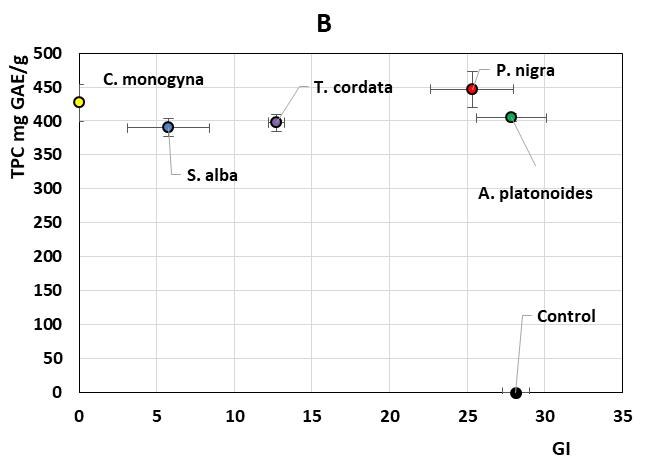


**(1)**


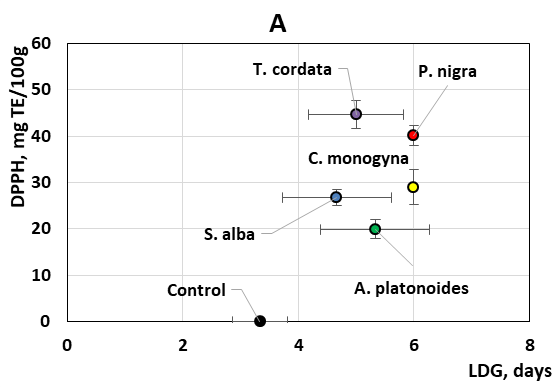

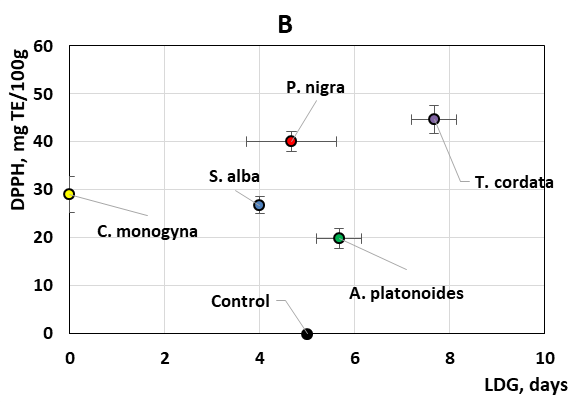


**
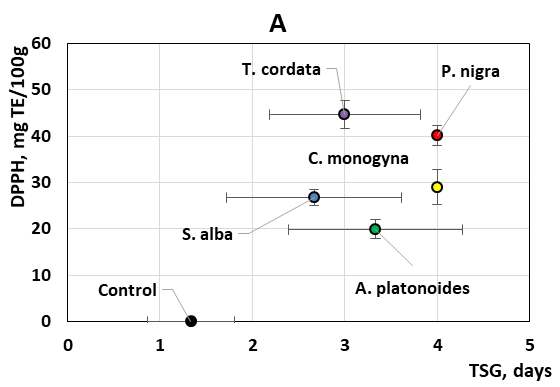

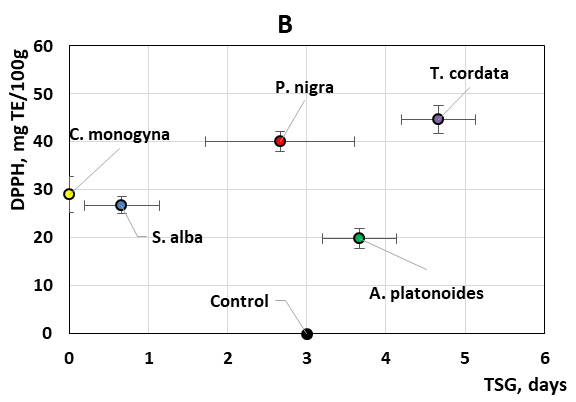
**

**(2)**

**
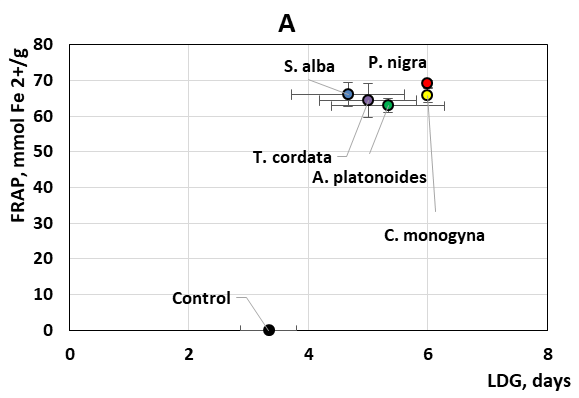

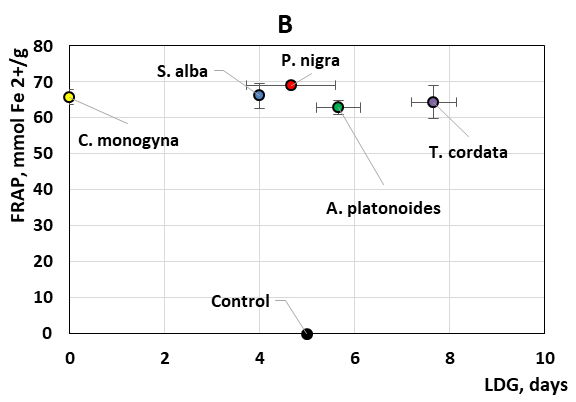
**

**
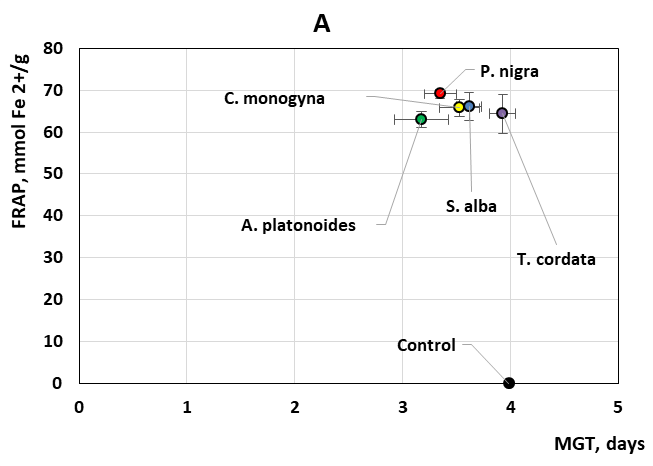

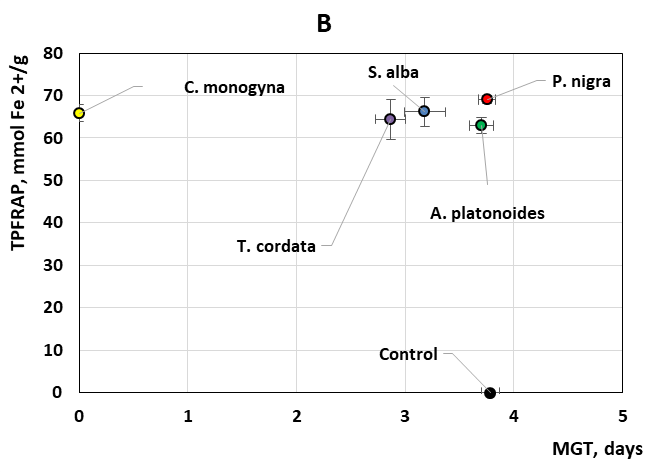
**

**(3)**

**Figure S5.** Dependence of TPC contents (1) and antioxidant activity (DPPH (2) and FRAP (3)) in mistletoe aqueous extracts, obtained from different host trees, on the germination speed of *Cucurbita maxima* ‘Hokkaido Orange’ test seeds treated for 2 hours (A) and 24 hours (B) before sowing
